# Comparison of strip meniscometry and Schirmer tear test results and tear film breakup time between healthy dogs and dogs with dry eye disease

**DOI:** 10.5935/0004-2749.20230057

**Published:** 2023

**Authors:** Felipe Franco Nascimento, João Victor Goulart Consoni Passareli, Luís Felipe da Costa Zulim, Danielle Alves Silva, Rogerio Giuffrida, Giovana José Garcia Estanho, Mayra Claudino Villa, Silvia Franco Andrade

**Affiliations:** 1 Program of Animal Science, Universidade do Oeste Paulista, Presidente Prudente, SP, Brazil.; 2 Faculty of Veterinary Medicine, Presidente Prudente, SP, Brazil.; 3 Department of Ophthalmology, Veterinary Hospital, Universidade do Oeste Paulista, Presidente Prudente, SP, Brazil.

**Keywords:** Keratoconjunctivitis sicca, Schirmer tear test, Strip meniscometry test, Tear film breakup time, Dogs, Ceratoconjuntivite seca, Teste lacrimal de Schirmer, Teste de meniscometria de tira, Tempo de ruptura do filme lacrimal, Cães

## Abstract

**Purpose:**

To compare strip meniscometry and Schirmer tear test 1 results and tear film breakup time between dogs with normal eyes and dogs diagnosed with keratoconjunctivitis sicca. Methods: One-hundred fifty-six eyes of 78 dogs, 88 normal eyes, and 68 eyes diagnosed with keratoconjunctivitis sicca were included in the study. The tests were performed in the following sequence: Schirmer tear test 1 was used to allocate the dogs to the normal or keratoconjunctivitis sicca group, followed by the strip meniscometry test and tear film breakup time measurement.

**Results:**

The results (mean ± standard deviation) of the tests in the normal group were as follows: Schirmer tear test 1, 22.75 ± 3.88 mm/min; strip meniscometry test, 10.01 ± 2.35 mm/5 sec; and tear film breakup time, 25.82 ± 5.47 sec. In the keratoconjunctivitis sicca group, the results were as follows: Schirmer tear test 1, 6.10 ± 4.44 mm/min; strip meniscometry test, 3.03 ± 2.62 mm/5 sec; and tear film breakup time, 10.78 ± 4.23 sec. The Spearman correlation coefficient in the keratoconjunctivitis sicca group was very high, with a significant difference between the strip meniscometry test and Schirmer tear test 1 (r=0.848, p<0.001), and moderate and significant between the strip meniscometry test and tear film breakup time (r=0.773, p<0.001). The cutoff for the strip meniscometry test for keratoconjunctivitis sicca was 7.0 mm/5 sec. Keratoconjunctivitis sicca was suspected when the values were <10 mm/5 sec.

**Conclusions:**

This study provided strip meniscometry test values from dogs with normal eyes and eyes with keratoconjunctivitis sicca, with high sensitivity and specificity compared with those of the Schirmer tear test 1. In the future, the strip meniscometry test may be another important quantitative test and could complement the gold standard Schirmer tear test for the diagnosis of keratoconjunctivitis sicca in dogs, an excellent animal model for the study of the disease.

## Abstract (pt)

**Objetivo:**

Comparar o teste de meniscometria em tira com o teste lacrimal de Schirmer 1 e o tempo de ruptura do filme lacrimal, em cães com olhos normais e cães com diagnóstico de ceratoconjuntivite seca.

**Métodos:**

Cento e cinquenta e seis olhos de 78 cães, 88 olhos normais e 68 olhos com diagnóstico de ceratoconjuntivite seca. Os testes foram realizados na seguinte sequência: o teste lacrimal de Schirmer 1 foi utilizado para alocar os cães no grupo normal ou no grupo ceratoconjuntivite seca, seguido pelo teste de meniscometria em tira e tempo de ruptura do filme lacrimal.

**Resultados:**

As médias e desvios-padrão para os olhos normais foram: teste lacrimal de Schirmer 1 = 22,75 ± 3,88 mm / min; teste de meniscometria em tira = 10,01 ± 2,35 mm / 5 seg; tempo de ruptura do filme lacrimal = 25,82 ± 5,47 seg; e para os olhos do grupo ceratoconjuntivite seca foram: teste lacrimal de Schirmer 1 = 6,10 ± 4,44 mm / min; teste de meniscometria em tira = 3,03 ± 2,62 mm / 5 seg; tempo de ruptura do filme lacrimal = 10,78 ± 4,23 seg. O teste de correlação de Spearman no grupo ceratoconjuntivite seca foi muito alto, com diferença significativa entre teste de meniscometria em tira e teste lacrimal de Schirmer 1 (r=0,848, p<0,001), moderada e significativa entre teste de meniscometria em tira e tempo de ruptura do filme lacrimal (r=0,773, p<0,001). O cut-off para teste de meniscometria em tira para ceratoconjuntivite seca foi identificado em 7,0 mm / 5 seg, valores abaixo de 10 mm / 5 seg podem ser considerados suspeitos para KCS. **Conclusões:** Este estudo forneceu valores de teste de meniscometria em tira em olhos normais e com ceratoconjuntivite seca em cães, revelando alta sensibilidade e especificidade em comparação com o teste lacrimal de Schirmer 1. No futuro, o teste de meniscometria em tira pode ser outro teste quantitativo importante e pode complementar o teste lacrimal de Schirmer padrão ouro para o diagnóstico de ceratoconjuntivite seca em cães, um excelente modelo animal para o estudo de ceratoconjuntivite seca.

## INTRODUCTION

Keratoconjunctivitis sicca (KCS), also known as dry eye syndrome or disease, is a chronic inflammatory disease that is predominantly immune mediated and is commonly diagnosed in both dogs and humans. Thus, dogs are an excellent animal model for research on this disease. KCS results in an insufficient production of the aqueous layer of the tear film (quantitative deficiency) or in excessive tear evaporation (qualitative deficiency) due to inadequate lipid or mucin layer production, which diminishes the protective function of the tear film^(1-5)^.

The diagnosis is based on clinical signs and the results of specific ophthalmic tests. The gold standard test to quantitatively measure the aqueous portion of the tear film is the Schirmer tear test (STT). However, other tests may be used in addition to STT to detect tear disorders, including the following: tear film breakup time (TBUT) measurement, which is a qualitative test used to assess evaporative dry eye disease and detect deficiencies in the mucin and lipid layers of the tear film; the phenol red test (PRT), which quantifies the aqueous tear film in 15 seconds; ocular surface staining, such as the lissamine green test (LGT) and Rose Bengal test (RBT), which stain damaged and devitalized cells in the conjunctiva and corneal epithelium; and conjunctival cytology, which is used to assess goblet cells, mucin production, lipid production, and meibomian gland function^(1,4,6-11)^.

Diagnostic tests for KCS should combine high precision, good sensitivity, and practicality^(12-16)^. Therefore, in humans, the need to develop a faster, less invasive, and easier-to-use method for the assessment of tear volume has led researchers to develop a simple and innovative method called the strip meniscometry test (SMT)^(16-18)^. The SMT was recently introduced in veterinary medicine to measure tear volume using a simple, fast (5 sec), and less invasive method, as the tip of the strip is projected to touch only the lower tear meniscus instead of inserting it in the conjunctival sac as in the Schirmer tear test. It has been recognized as a promising technique, particularly in screening and diagnosing disorders of the ocular surface, such as KCS.

Another advantage of the SMT is the lower volume of tears absorbed by the strips, which allows other tests to be conducted shortly afterward^(19-21)^. The lacrimal lake provides a reservoir that contributes to the formation of the preocular tear film with each blink, accommodates excess tears during reflex tearing, and contains 75% to 90% of the aqueous tear volume, which positively correlated with the lacrimal secretory rate^(16)^. The lacrimal lake volume has been reported to be reduced in tear-deficient dry eye disease, and its measurement is of great value in the diagnosis of the disease. In humans, using the SMT in tandem with other ocular surface tests such as STT has been shown to promote higher specificity than using the SMT alone^(16-18)^.

Recently, a study that included 621 dogs with normal eyes and KCS^(21)^ described the clinical correlations among the SMT, STT, and PRT results. The study revealed that the SMT results better agreed with the STT results than with the PRT results. The cutoff for the SMT was 10 mm/5 sec to discriminate normal eyes from tear-deficient eyes. The authors concluded that the SMT could be superior to PRT in discriminating tear-deficient eyes and could be useful as an initial diagnostic tool to rule out normal eyes with a short testing time.

To date, no study has compared the SMT with the TBUT in dogs, an excellent animal model for the study of KCS. Thus, the aim of this study was to assess the SMT and its results in relation to STT-1 and TBUT in dogs with normal eyes and dogs with eyes diagnosed with KCS. In addition, we determined its cutoff value, sensitivity, and specificity on the basis of a receiver-operating characteristic (ROC) curve.

## METHODS

### Animals

The study was conducted in accordance with the standards for animal experimentation of the UNOESTE Ethical Committee on Animal Use (protocol No. 3895) and the ARVO (Association for Research in Vision and Ophthalmology) guidelines for the use of animals in ophthalmic and visual research.

To determine the minimum sample size required to estimate the mean tear production using the SMT, we used the formula described by Pagano and Gauvreau^(22)^, with a standard deviation value based on the results obtained by Rajaei et al.^(19)^. On the basis of these parameters, we concluded that a minimum of 50 normal eyes and 50 eyes with KCS would be needed. The actual sample sizes used in the study were larger to improve reliability, and 156 eyes from 78 dogs were assessed and classified into different groups according to the STT-1 results, with 88 in the normal group (STT-1 ≥15 mm/min) and 68 in the KCS group. The STT-1 results were interpreted as follows: “severe” 0-5 mm/min (n=32); “moderate” 6-10 mm/min (n=22); and “subclinical” 11-14 mm/min (n=14). Of the dogs, 30 were male (14 castrated and 16 intact) and 48 were female (30 castrated and 18 intact), with ages ranging from 3 months to 16 years and a mean weight of 11.05 ± 7.13 kg. The sex, age, and breed of the dogs in each group are described in table 1. All the animals included in the study were evaluated at the outpatient clinic of the Ophthalmology Department of the Veterinary Hospital of UNOESTE, Presidente Prudente, SP, Brazil.

**Table 1:** Sex, age in years, and breed (most to least prevalent) of the dogs in each group according to STT classification (mm/min) that were evaluated at the Veterinary Hospital of UNOESTE, Presidente Prudente, SP, Brazil

Group	Sex n(%)	Age mean ± standard deviation (range values)	Breed
**Normal**(STT **≥**5) n=44	33 males (75) 11 females (25)	3.0±1.6(1-6)	French bulldog (15); mixed breed (13); Lhasa Apso (4); English bulldog (3); Shih-tzu (2); Yorkshire Terrier (2); Boxer (1); Campero bulldog (1); Chihuahua (1); Cocker Spaniel (1); and Maltese (1)
**KCS Severe**(STT 0-5) n=16	6 males (37,5) 10 females (62,5)	9.1±3.5*(4-16)	Poodle (3); Lhasa Apso (3); Yorkshire Terrier (2); Cocker Spaniel (2); Shih-tzu (1); Pinscher (2); Pekinese (1); Chow Chow (1); and mixed breed (1)
**KCS Moderate**(STT 6-10) n=11	2 males (18,2) 9 females (81,8)	7.8±2.7*(4-12)	Shih-tzu (4); Poodle (3); English Bulldog (2); Lhasa Apso (1); and Yorkshire Terrier (1)
**KCS** Subclinical (STT 11-14) n=7	4 males (57,1) 3 females (42,9)	5.1±2.6 (0,5-8)	Miniature Schnauzer (2); mixed breed (2); Lhasa Apso (1); Maltese (1); and Rottweiler (1)

* *p*<0.05 (Tukey test to compare age with the normal group).

### Ophthalmic examinations

All the animals included in the study had initial examinations performed 1 day before the specific tear tests. Ocular clinical signs were assessed with a portable slit lamp (SL-15, Kowa, Japan). The pupillary light reflex (PLR) test with a punctiform light, direct ophthalmoscopy of the fundus (Pocket Jr, Welch Allyn, USA), rebound tonometry (Tonovet, Icare, Finland) to verify the intraocular pressure, and fluorescein test (Fluoresceína; Allergan, Brazil) for staining the corneal ulcers were also performed. One day after these examinations, specific tear tests were performed to diagnose KCS and assess the ocular surface in the following order: the STT-1, SMT, and TBUT. The inclusion criteria for the study were as follows: for healthy eyes, a STT-1 result of ≥15 mm/min and a normal ophthalmic examination result, and for eyes with KCS, a STT-1 result ≤14 mm/min, classified as follows: severe (0-5 mm/min), moderate (6-10 mm/min), and subclinical (11-14 mm/min). The exclusion criteria were increased intraocular pressure, corneal ulcer, negative pupillary reflex, and abnormalities in the eye fundus. All ocular tests and examinations were performed by the same examiner (FFN). All the measurements were scheduled and performed between 8:00 am and 12:00 pm.

STT-1 was conducted without a topical anesthetic by introducing 0.5 cm of the filter paper strip (Schirmer Tear Test Ophthalmos, Brazil) to the conjunctival sac for 1 min. The paper strip was then removed, and the wet area was immediately measured (ignoring the 0.5-cm portion). The animals were considered positive for KCS when the STT-1 result was ≤14 mm/min, and they were considered healthy when the STT-1 result was ≥15 mm/min and the results of the ophthalmic examinations were normal^(11)^.

**Figure 1:**
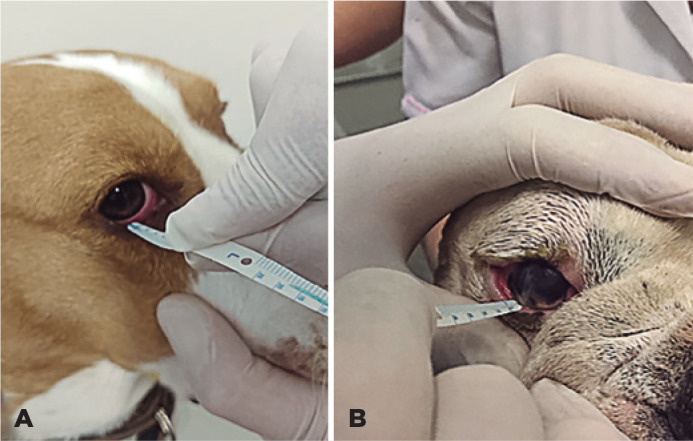
(A) Strip meniscometry test performed on a dog with a normal eye. (B) Strip meniscometry test performed on a dog with keratoconjunctivitis sicca.

The SMT was conducted by placing the tip of the paper strip (I-Tear; I-Med Pharma, Canada) at the inferior tear meniscus for 5 sec (Figure 1) without touching the eyelid or ocular surface and observing when tears entered the ridge and turned blue (when they come in contact with the natural blue dye at the tip of the strip) without expanding onto the hydrophobic pellicle to the sides. After the time was up, the value obtained was immediately recorded^(19-21)^.

The TBUT was performed by applying 1 drop of 1% fluorescein eye drops (Fluoresceína; Allergan, Brazil). After two blinks, the eyelid was held open manually, and the cornea was observed with a portable slit lamp (SL-15; Kowa, Japan) with cobalt blue illumination. The tear film breakup time (appearance of the first dry spots on the cornea) was measured in seconds, and times ≥20 sec were considered normal^(10,11)^.

### Statistical analysis

The Shapiro-Wilk test was performed to validate the assumption of normal distributions of STT-1, SMT, and TBUT data. The Spearman correlation analysis was used to evaluate the relationships between the STT-1, SMT, and TBUT results. To assess the presence of significant differences among the KCS STT-classified Groups, STT-1, SMT, and TBUT results and age were compared using one-way analysis of variance with Tukey method contrasts. ROC curves were constructed to obtain the areas under the curve (AUC) of the SMT and TBUT results in reference to the STT-1-classified Groups. Sensitivity and specificity were also calculated as functions of the cutoff SMT score. To determine the ideal cutoff point for balancing sensitivity and specificity, the Youden index (*J*) was calculated using the formula, sensitivity + specificity - 1. All calculations were conducted using the R software (The R Foundation for Statistical Computing, 2018), with a significance level of 5%.

## RESULTS

The sex, age, and breed of the dogs included in the study are described in table 1. A higher percentage of males were included in the normal group and subclinical KCS Group, and a higher percentage of females were included in the severe and moderate KCS Groups. We found a significant difference (p<0.05) in age between the severe and moderate KCS Groups (more advanced ages) and the normal group. The ophthalmic examination results from the STT-1, SMT, and TBUT are described in table 2. Significant differences (p<0.05) in all the KCS ranges in STT-1, SMT and TBUT examinations were found in the normal Group. In the STT-classified eyes, the highest percentage observed was with score 1, that is, 12.2% in severe KCS, 18.2% in moderate KCS, and 15.4% in subclinical KCS.

**Table 2:** Results of the ophthalmic examinations in the STT-1, SMT, and TBUT (mean ± standard deviation); medians and ranges obtained from the Schirmer tear test 1 (STT-1) in mm/min; strip meniscometry test (SMT) results in mm/5 sec; and tear film breakup test (TBUT) results in seconds

Tests	Total eyes (n=156)	STT classified (mm/min)				
		Normal (≥15) n=88	KCS severe (0-5) n=32	KCS moderate (6-10) n=22	KCS subclinical (11-14) n=14	KCS total (≤14) n=68
**STT-1 (mm/min)**						
**Mean ± SD**	15.49 ± 9.25	22.75 ± 3.88 (a)	2.21 ± 2.03 (b)	8.27 ± 1.52 (c)	12.62 ± 0.96 (d)	6.10 ± 4.44
**Median**	18	23	2	9	13	6
**Range**	0-30	15-30	0-5	6-11	11-14	0-14
**SMT (mm/5 sec)**						
**Mean ± SD**	6.97 ± 4.26	10.01 ± 2.35 (a)	1.21 ± 1.71 (b)	4.40 ± 1.97 (b)	5.60 ± 2.40 (c)	3.03 ± 2.62
**Median**	8	9	0	5	5	3
**Range**	0-19	8-19	0-8	0-8	2-10	0-10
**TBUT (sec)**						
**Mean ± SD**	19.26 ± 8.97	25.82 ± 5.47 (a)	8.21 ± 3.30 (b)	12.32 ± 3.12 (b)	15.46 ± 2.82 (c)	10.78 ± 4.23
**Median**	20	24	9	13	16	11
**Range**	3-44	20-44	3-15	5-16	9-19	3-19

Means followed by the same letter in the same line do not differ in the Tukey post hoc test.

The Spearman correlation coefficients between the results of the ophthalmic tests (STT-1, SMT, and TBUT) are described in table 3. Strong and significant correlations (p<0.05) were found among the SMT and STT results; and moderate and significant correlations, between the TBUT results for the STT1-classified measurement ranges of 0-5 and 6-10 mm/min. However, no significant correlation (p>0.05) was observed between these parameters for the range 11-14 mm/min. A significant correlation (p<0.05) was found between the SMT and TBUT for the 0-5 mm/min range. In the normal STT1-classified range, a significant correlation was found between STT and TBUT. Considering the overall results, including all ranges of the STT1-classified KCS values, the Spearman correlation in the KCS Groups was very high and significant between the SMT and STT-1 Groups (r=0.848, p<0.001), moderate and significant between the SMT and TBUT Groups (r=0.773, p<0.001), and very low and not significant between the SMT and LGT Groups (r=-0.098, p=0.424).

**Table 3:** Spearman correlation coefficients between the different ophthalmic tests

STT1-classified*	Correlation	ρ**	95% CI	p value** *
**Normal**	STT-1 x SMT	0.146	-0.065 to 0.345	0.173
	STT-1 x TBUT	0.228	0.020 to 0.418	0.032
	SMT x TBUT	0.043	-0.168 to 0.250	0.690
**0-5**	STT-1 x SMT	0.681	0.440 to 0.830	<0.0001
	STT-1 x TBUT	0.353	0.011 to 0.621	0.043
	SMT x TBUT	0.430	0.101 to 0.673	0.012
**6-10**	STT-1 x SMT	0.480	0.076 to 0.751	0.023
	STT-1 x TBUT	0.061	-0.370 to 0.470	0.786
	SMT x TBUT	0.440	0.022 to 0.726	0.040
**11-**14	STT-1 x SMT	0.183	-0.408 to 0.667	0.548
	STT-1 ´ TBUT	0.317	-0.283 to 0.740	0.290
	SMT x TBUT	0.460	-0.121 to 0.806	0.113
**KCS total**	STT-1 x SMT	0.848	0.700 to 0.887	<0.0001
	STT-1 x TBUT	0.846	0.794 to 0.885	<0.0001
	SMT x TBUT	0.773	0.701 to 0.830	<0.0001

* STT-1 classification: normal healthy eyes, STT-1 ≥15 mm/min; severe KCS, 0-5mm/min; moderate KCS (6-10 mm/min); and subclinical KCS, 11-14 mm/min.

** Spearman correlation coefficient; 95% CI, estimation of *r* through an interval with 95% confidence.

*** Significance level for the hypothesis that ρ differs significantly from 0.

**Figure 2:**
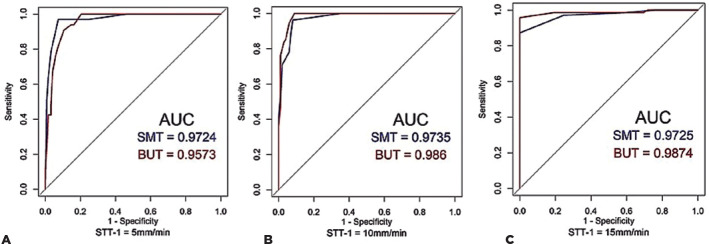
Receiver-operating characteristic (ROC) curves of the strip meniscometry test (SMT) and tear film breakup time (TBUT) measurement for different STT-1 cutoff criteria: (A) 5, (B) 10, and (C) 15 mm/min. The area under the curve (AUC) is an important measure to assess the predictive accuracy of a test. These values for the SMT and TBUT are inset in each plot. The sensitivity, specificity, and Youden index associated with the cutoff points for the SMT are correspondingly reported in table 4.

The sensitivity, specificity, and Youden index (*J*) values based on the STT-1 cutoff criteria (i.e., 5, 10, and 15 mm/min) are shown in table 4. The ROC curve showing the cutoff value for the SMT is shown in figure 2. A strong correlation was found between the SMT results and those of the STT-1 and TBUT.

**Table 4:** Sensitivity, specificity, and Youden index (*J*) values based on each STT-1 cutoff criteria (i.e., 5, 10, and 15 mm/min)

SMT cutoff (mm/5 sec)	STT-1 criterion								
	5 mm/min			10 mm/min			15 mm/min		
	Sensitivity	Specificity	J	Sensi-tivity	Specificity	J	Sensitivity	Specificity	J
**<1**	0.98	0.64	0.62	1.00	0.42	0.42	1.00	0.32	0.32
**<2**	0.97	0.79	0.76	0.99	0.53	0.52	1.00	0.42	0.42
**<3**	0.93	0.97	0.90	0.98	0.71	0.69	1.00	0.58	0.58
**<4**	0.86	0.97	0.83	0.94	0.78	0.72	1.00	0.69	0.69
**<5**	0.80	0.97	0.77	0.93	0.89	0.82	1.00	0.79	0.79
**<6**	0.76	0.97	0.73	0.92	0.96	0.88	1.00	0.86	0.86
**<7**	0.76	0.97	0.73	0.91	0.96	0.87	1.00	0.87	0.87
**<8**	0.54	1.00	0.54	0.65	1.00	0.65	0.75	0.97	0.72
**<9**	0.29	1.00	0.29	0.36	1.00	0.36	0.41	0.99	0.40
**<10**	0.17	1.00	0.17	0.21	1.00	0.21	0.25	1.00	0.25
**<11**	0.16	1.00	0.16	0.20	1.00	0.20	0.24	1.00	0.24
**<12**	0.12	1.00	0.12	0.15	1.00	0.15	0.18	1.00	0.18
**<13**	0.07	1.00	0.07	0.09	1.00	0.09	0.11	1.00	0.11
**<14**	0.04	1.00	0.04	0.05	1.00	0.05	0.06	1.00	0.06
**<15**	0.02	1.00	0.02	0.02	1.00	0.02	0.02	1.00	0.02
**<16**	0.02	1.00	0.02	0.02	1.00	0.02	0.02	1.00	0.02
**<17**	0.02	1.00	0.02	0.02	1.00	0.02	0.02	1.00	0.02
**<18**	0.01	1.00	0.01	0.01	1.00	0.01	0.01	1.00	0.01
**<19**	0.00	1.00	0.00	0.00	1.00	0.00	0.00	1.00	0.00
**<20**	0.00	1.00	0.00	0.00	1.00	0.00	0.00	1.00	0.00

*J* (Younden index); SMT (strip meniscometry test); STT (Schirmer tear test).

## DISCUSSION

This is the first study in dogs that compared the usefulness of the SMT with that of the main ophthalmic tests for the diagnosis of KCS, including the gold standard STT-1 and TBUT. A limitation of our study was that the number of KCS cases (n=34) was lower than that of normal dogs (n=44). The study was conducted in Presidente Prudente, an interior city in the state of São Paulo, with a population of around 200,000 inhabitants and a smaller dog population than the large cities. This resulted in greater difficulty in obtaining a larger number of KCS cases.

Breed, sex, and age predisposition to KCS in dogs have been proposed by several reports in the literature^(1,3,8,21,23-26)^. The English Bulldog, Lhasa Apso, Shih-Tzu, Poodle, West Highland White Terrier, and Cocker Spaniel are recognized worldwide as predisposed breeds^(1,3,21,24-26)^. In our study, the most prevalent breeds diagnosed with KCS were the Poodle (n=6), Lhasa Apso (n=5), Shi Tzu (n=5), and Yorkshire Terrier (n=3). A female predisposition to KCS has been reported by several authors^(21,25)^, probably due to hormonal changes^(25)^, which was also observed in our study, with more females diagnosed with KCS in the severe to moderate KCS Groups. The ages of the animals with severe to moderate KCS were significantly more advanced (p<0.05) than those in the normal and subclinical KCS Groups, which agrees with others authors from the human^(26)^ and veterinary literature^(21,25,26)^. In aging eyes, risk factors such as polypharmacy, androgen deficiency, decreased blink rates, and oxidative stress can predispose the patient to developing dry eyes that is frequently more severe^(26)^.

In our study, the values were similar to those described in other studies in dogs^(19,21)^. Similar studies in humans^(27)^ reported SMT values of 6.4 ± 2.0 mm/5 sec in healthy eyes and 4.8 ± 1.6 mm/5 sec in KCS eyes. A study in capuchin monkeys^(20)^ obtained a median and semi-interquartile range for the SMT of 7.5 ± 1.85 mm/5 sec for the right eye and 8.5 ± 1.62 mm/5 sec for the left eye. The values observed in humans with KCS were also similar to the results observed in this study (3.02 ± 2.52 mm/5 sec). These findings are in line with the observations of another study^(18)^ that concluded that the measurements of the tear meniscus were significantly lower in patients with KCS than in healthy patients.

STT-1 has been used in both humans and animals as the gold standard for the quantitative diagnosis of KCS by measuring the volume of the aqueous portion of the tear film. However, the test requires patients to endure the discomfort caused by inserting the paper strip in the eye, which characterizes the technique as invasive^(12-15)^. The SMT is a noninvasive diagnostic tool for KCS that can be performed in 5 sec without inducing the lacrimation reflex. However, the technique requires a higher precision than STT-1 because it does not touch the cornea or conjunctiva during the examination^(16-21)^.

Combining the SMT with other lacrimal function tests, particularly the TBUT, results in acceptable sensitivity and specificity, which is valuable in the assessment of ocular surface diseases^(16)^. This study shows that the mean ± standard deviation values for the TBUT in dogs was 25.82 ± 5.47 sec (range, 20-44 sec) for normal eyes and 10.78 ± 4.23 (range, 3-19 sec) for KCS eyes. This confirms the observation of Seyer et al.^(28)^ that the TBUT values decrease as the severity of the corneal surface disease increases.

In the Spearman correlation analysis, we observed that the SMT and STT-1 results showed a strong correlation with the total number of KCS cases (r=0.848, n=68), likely due to the larger population sample and STT-1 classification in the range of 0-5 mm/min (r=0.681, n=33). Meanwhile, a moderate correlation was observed between the SMT and TBUT results and the total number of KCS cases (r=0.773, n=68). These findings are in agreement with the study of Miyasaka et al.^(21)^, who reported an increasing agreement between SMT and STT results and increasing severity of tear deficiency.

By observing the ROC curves for the different degrees of severity, we verified that the SMT presents higher accuracy, denoted by the AUC, when using a cutoff point of 10 mm/5 sec (Figure 2). Table 4 shows that the SMT cutoff points with the best classification power denoted by *J* values were 3, 6, and 7 mm/5 sec for the STT-1-classified references of 5, 10, and 15 mm/min, respectively. From these values, we can use as a cutoff value for SMT of 7 mm/5 sec for our region to predict positivity for KCS, with a sensitivity and specificity of 76% and of 97% for “severe” 91% and 96% for “moderate” and 100% and 87% for “subclinical” respectively (Table 4). In our study, the mean and standard deviation, median, and range for the normal eyes were 10.01 ± 2.35 mm/5 sec, 9 mm/5 sec, and 8-19 mm/5 sec, respectively. Thus, values <10 mm/5 sec may be considered a basis to suspect KCS in our region and may serve as parameters for other studies.

A study in humans^(27)^ defined an ideal cutoff for the SMT of ≤5 mm/5 sec, resulting in a sensitivity of 70.6% and specificity of 84.6% as a first-screening tool to rule out KCS, with a sensitivity of 0.96 for “severe”, 0.91 for “moderate”, and 0.73 for “subclinical” states.

In conclusion, the SMT is an examination that is fast and easy to perform in dogs, with distinct values between healthy and KCS eyes. The SMT presents excellent sensitivity and specificity compared with STT-1, to which it presented a strong correlation. In the future, the SMT may be another important quantitative test and could compliment the gold standard STT in the diagnosis of KCS in dogs, an excellent animal model for the study of KCS.
